# A Kinetic Model of Dopamine- and Calcium-Dependent Striatal Synaptic Plasticity

**DOI:** 10.1371/journal.pcbi.1000670

**Published:** 2010-02-12

**Authors:** Takashi Nakano, Tomokazu Doi, Junichiro Yoshimoto, Kenji Doya

**Affiliations:** 1 Graduate School of Information Science, Nara Institute of Science and Technology, Ikoma, Japan; 2 Okinawa Institute of Science and Technology, Uruma, Japan; 3 Osaka Bioscience Institute, Suita, Japan; École Normale Supérieure, College de France, CNRS, France

## Abstract

Corticostriatal synapse plasticity of medium spiny neurons is regulated by glutamate input from the cortex and dopamine input from the substantia nigra. While cortical stimulation alone results in long-term depression (LTD), the combination with dopamine switches LTD to long-term potentiation (LTP), which is known as dopamine-dependent plasticity. LTP is also induced by cortical stimulation in magnesium-free solution, which leads to massive calcium influx through NMDA-type receptors and is regarded as calcium-dependent plasticity. Signaling cascades in the corticostriatal spines are currently under investigation. However, because of the existence of multiple excitatory and inhibitory pathways with loops, the mechanisms regulating the two types of plasticity remain poorly understood. A signaling pathway model of spines that express D1-type dopamine receptors was constructed to analyze the dynamic mechanisms of dopamine- and calcium-dependent plasticity. The model incorporated all major signaling molecules, including dopamine- and cyclic AMP-regulated phosphoprotein with a molecular weight of 32 kDa (DARPP32), as well as AMPA receptor trafficking in the post-synaptic membrane. Simulations with dopamine and calcium inputs reproduced dopamine- and calcium-dependent plasticity. Further *in silico* experiments revealed that the positive feedback loop consisted of protein kinase A (PKA), protein phosphatase 2A (PP2A), and the phosphorylation site at threonine 75 of DARPP-32 (Thr75) served as the major switch for inducing LTD and LTP. Calcium input modulated this loop through the PP2B (phosphatase 2B)-CK1 (casein kinase 1)-Cdk5 (cyclin-dependent kinase 5)-Thr75 pathway and PP2A, whereas calcium and dopamine input activated the loop via PKA activation by cyclic AMP (cAMP). The positive feedback loop displayed robust bi-stable responses following changes in the reaction parameters. Increased basal dopamine levels disrupted this dopamine-dependent plasticity. The present model elucidated the mechanisms involved in bidirectional regulation of corticostriatal synapses and will allow for further exploration into causes and therapies for dysfunctions such as drug addiction.

## Author Summary

Recent brain imaging and neurophysiological studies suggest that the striatum, the start of the basal ganglia circuit, plays a major role in value-based decision making and behavioral disorders such as drug addiction. The plasticity of synaptic input from the cerebral cortex to output neurons of the striatum, which are medium spiny neurons, depends on interactions between glutamate input from the cortex and dopaminergic input from the midbrain. It also links sensory and cognitive states in the cortex with reward-oriented action outputs. The mechanisms involved in molecular cascades that transmit glutamate and dopamine inputs to changes in postsynaptic glutamate receptors are very complex and it is difficult to intuitively understand the mechanism. Therefore, a biochemical network model was constructed, and computer simulations were performed. The model reproduced dopamine-dependent and calcium-dependent forms of long-term depression (LTD) and potentiation (LTP) of corticostriatal synapses. Further *in silico* experiments revealed that a positive feedback loop formed by proteins, the protein specifically expressed in the striatum, served as the major switch for inducing LTD and LTP. This model could allow us to understand dynamic constraints in reward-dependent learning, as well as causes and therapies of dopamine-related disorders such as drug addiction.

## Introduction

**Figure 1:**
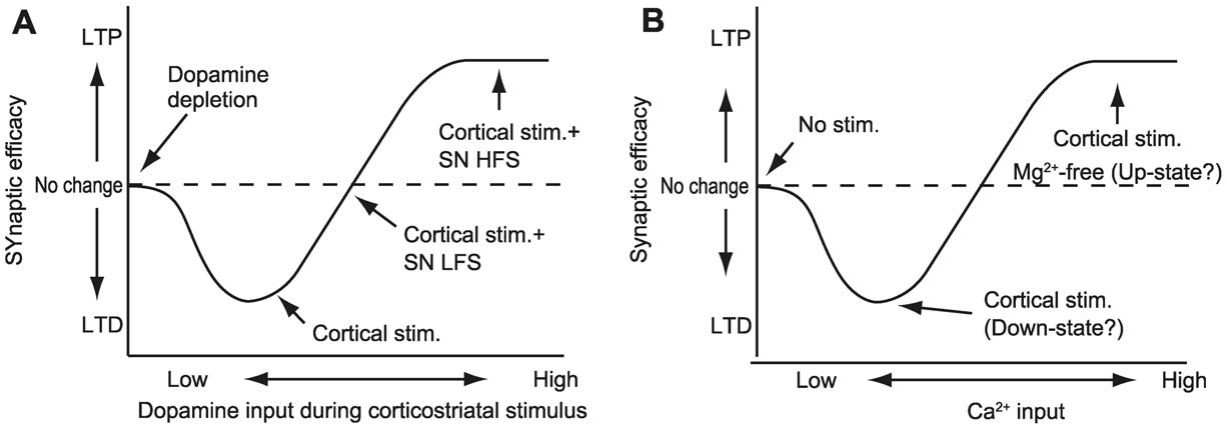
Schematic diagrams of dopamine- and calcium-dependent synaptic plasticity. (A) Dopamine-dependent synaptic plasticity (modified from [27]). (B) Calcium-dependent synaptic plasticity. The abbreviations used in superimposition are as follows: SN - substantia nigra; LFS - low-frequency stimulation; and HFS - high-frequency stimulation. The altered direction of synaptic efficacy depends on input intensity of dopamine and calcium.

The basal ganglia integrates sensory and motivational signals to achieve goal-directed actions and cognition [1]–[3]. The striatum, the input site of the basal ganglia, receives glutamatergic input from the cortex and dopaminergic input from the substantia nigra and the ventral tegmental area. Dopaminergic input to the striatum plays a critical role in motor and cognitive control, as evidenced in Parkinson's disease and drug addiction [4]–[6]. Glutamatergic and dopaminergic fibers converge onto single synapses of medium spiny neurons [7], which are the striatal output neurons. Corticostriatal synapse efficacy is regulated by cortical glutamatergic input and dopaminergic input. While glutamatergic input without dopamine input results in long-term depression (LTD), coincident glutamatergic and dopaminergic inputs can cause long-term potentiation (LTP) [8],[9]. This dopamine-dependent plasticity is a critical element for linking sensory and cognitive inputs from the cortex with reward-related signals from firing dopaminergic neurons to establish goal-directed behaviors [2]. Furthermore, glutamatergic input in magnesium-free solution, which results in massive calcium influx through NMDA-type receptors, induces LTP without dopaminergic input. Therefore, corticostriatal synapses exhibit two types of plasticity: dopamine-dependent plasticity requiring co-activation of glutamatergic and dopaminergic inputs [9],[10] and calcium-dependent plasticity requiring only glutamatergic input [8],[11] (Fig. 1).

In the present study, a dynamic model of the intracellular signaling cascade, which links glutamatergic and dopaminergic inputs to regulation of glutamatergic receptors, was constructed to elucidate the dynamic molecular mechanisms behind the two types of corticostriatal synaptic plasticity. This model will provide a basis for understanding and predicting the effects of pharmacological manipulations and genetic variations on reward-dependent functions involving the basal ganglia, such as motor learning, cognitive control, and drug addiction.

The intracellular signaling cascade involved in synaptic regulation of corticostriatal synapses has been extensively studied [12]–[19]. Glutamatergic input increases intracellular calcium ion concentration, and dopaminergic input increases intracellular cyclic adenosine 3′,5′-monophosphate (cAMP) by activating adenylyl cyclase 5 (AC5). DARPP-32, the dopamine- and cyclic AMP-regulated phosphoprotein, with a molecular weight of 32 kDa, has multiple phosphorylation sites that are affected by calcium and cAMP. DARPP-32, in turn, regulates enzymes that influence phosphorylation of AMPA-type glutamate receptors. Insertion of phosphorylated AMPA receptors into the post-synaptic membrane is the main mechanism of glutamatergic synaptic plasticity [20]. Despite extensive knowledge of this system, multiple feedforward and feedback pathways, with excitatory and inhibitory interactions in the molecular network, results in complicated mechanisms that are difficult to comprehend with schematic diagrams or purely analytical methods. Therefore, quantitative computer simulations of the signaling cascade under various manipulations are required, such as blockades and knockouts, to understand the basic mechanism of the entire pathway and the roles of specific elements.

Existing simulation models have considered subnetworks of the signaling pathways surrounding DARPP-32. The model by Fernandez *et. al.* [21] considered intracellular calcium ion and cyclic AMP concentrations to be the inputs and simulated activation of three DARPP-32 phosphorylation sites. Lindskog *et. al.* [22] utilized intracellular calcium and D1-type dopamine receptor (D1R) binding as inputs and simulated activation of enzyme phosphorylation and de-phosphorylation. In addition, Barbano *et. al.* [23] analyzed a model making use of glutamate and dopamine for input, demonstrating the stability of the net state of DARPP-32 phosphorylation in the presence of noise. However, none of these models included the resulting phosphorylation of AMPA receptors, which is directly related to LTP and LTD. In addition, the models only focused on dopamine-dependent plasticity and did not consider the mechanisms of calcium-dependent plasticity.

In the present study, a complete model of the signal transduction pathway was constructed, with intracellular calcium ion and extracellular dopamine concentrations serving as inputs and post-synaptic membrane insertion of AMPA-type glutamate receptors for the output. The following was demonstrated *in silico*: 1) The model reproduced both calcium- and dopamine-dependent plasticity and determined the sub-pathways responsible for different types of plasticity. 2) The model predicted that the pathway through cyclin-dependent kinase 5 (Cdk5) is crucial for inducing synaptic depression with weak calcium input. 3) The model determined that a positive (double-negative) feedback loop, which included DARPP-32, plays an important role in LTP induction, with either strong calcium input or simultaneous calcium and dopamine inputs.

In the following sections of this manuscript, the neurobiological literature for building the transduction pathway model will be reviewed, followed by an explanation of the structure, computing method, and simulation input and output. Experimental results *in silico* demonstrated the following: the pathway response to different calcium and dopamine input levels, the effect of DARPP-32 knockout, and analysis of the positive feedback loop. The study concludes with the new knowledge gained by this simulation and directions for further studies based on this model.

### Neurobiology of corticostriatal synaptic plasticity

The present study reviews electrophysiological studies on corticostriatal synapse plasticity of medium spiny neurons and molecular biological studies focused on intracellular signaling cascades involved in this plasticity.

#### Dopamine-dependent synaptic plasticity

In corticostriatal slices or co-culture preparations, tetanic stimulation of cortical fibers inducing striatal cell firing results in long-term depression (LTD) of corticostriatal synapses [8],[24],[25]. In contrast, simultaneous stimulation of dopaminergic neurons in the substantia nigra during cortical stimulation results in long-term potentiation (LTP) with high frequency stimulation, and no change in synaptic efficacy at low frequency stimulation (i.e., levels corresponding to spontaneous firing) [9],[10],[26],[27]. In addition, under dopamine depletion, cortical stimulation does not alter corticostriatal synaptic efficacy [8]. Fig. 1A shows that cortical glutamatergic input can cause either LTD or LTP of corticostriatal synapses depending on the strength of simultaneous dopaminergic input.

#### Calcium-dependent synaptic plasticity

Cortical stimulation without dopamine input induces LTP of corticostriatal synapses. In slice preparations cultured in magnesium-free solutions, tetanic stimulation of cortical fibers induces LTP [11],[28],[29]. In anesthetized in vivo preparations or co-cultures, the resting membrane potential of medium spiny neurons alternates between an up-state of −60 mV and a down-state of −85 mV, with a low frequency of approximately 1 Hz. During the up-state, when magnesium inhibition of NMDA receptors is removed [30], tetanic stimulation of cortical fibers induces LTP in corticostriatal synapses [31]–[34]. Therefore, even with little or no dopamine, high levels of intracellular calcium, either through inotropic glutamate receptors and voltage-dependent calcium channels (VDCCs) or through endoplasmic reticulum (ER) calcium release via activation of metabotropic glutamate receptors (mGluRs), can revert LTD of corticostriatal synapses to LTP (Fig. 1B).

#### Intracellular signal transduction

**Figure 2:**
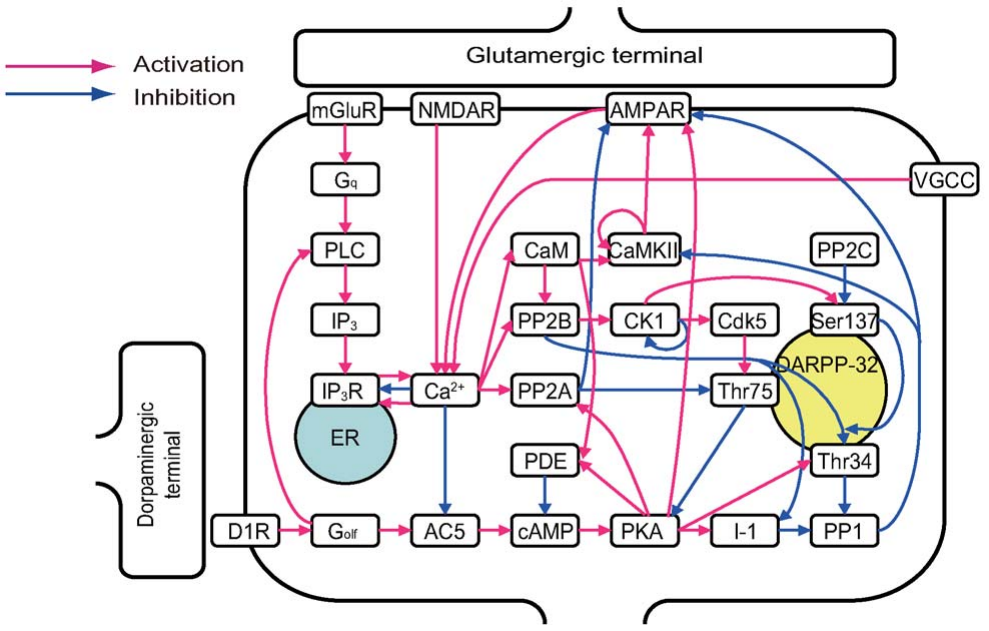
Block diagram of the signal transduction model in medium spiny neurons. The red and blue arrows indicate activation and inhibition, respectively. Detailed information on the regulatory pathways is provided in the Materials and Methods section, and the rough sketch of the signal flow is as follows. Glutamate binds to its corresponding receptors and increases intracellular calcium. D1R binding to dopamine increases cAMP. Calcium and cAMP alter the number of AMPA membrane receptors via downstream cascades and, thereby, regulate the synaptic efficacy of the neuron. The bi-directional effect of calcium on 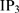 receptor should be mentioned. The activation level (open probability) of 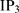 receptor displays a bell-shaped response curve to intracellular calcium concentrations. The 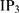 receptor activation level is maximal when intracellular calcium concentration is approximately 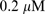 [107]. However, more (and less) calcium reduces 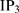 receptor activation. To represent this regulation, two complementary arrows represent activation and inhibition from calcium to 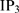 receptor in this diagram. In addition, one arrow originates from Ser137 and terminates at an arrow from PP2B to Thr34. Phosphorylation of Ser137 decreases the rate of Thr34 dephosphorylation by PP2B. Therefore, Ser137 contributes to disinhibition of the PP2B-Thr34 pathway [55]. The arrow from Ser137 represents this effect.

The intracellular signaling cascades that regulate synaptic efficacy of the corticostriatal synapse have been extensively studied [35]–[39]. Medium spiny neurons are divided into two subclasses: those expressing D1Rs, which project to the basal ganglia output nucleus (reticular part of the substantia nigra and internal segment of the globus pallidus), and those expressing D2-type dopamine receptors (D2Rs), which project to the external segment of the globus pallidus [40],[41]. The present study modeled D1R-expressing neurons based on previous literature and databases [42]. Fig. 2 shows the summary block diagram of the signaling cascade model. The model details are provided in Materials and Methods.

## Materials and Methods

### Mathematical formulation

All signaling pathway reactions shown in Fig. 2 are represented by binding and enzymatic reactions.

Binding reaction of molecule A and molecule B to form molecule AB
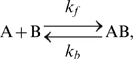
(1)where 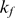 and 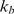 are rate constants for forward and backward reactions, is simulated by the ordinary differential equation:
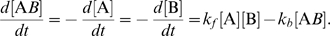
(2)The rate constants 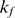 and 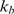 were related to the dissociation constant 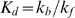 and the time constant 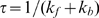, i.e., 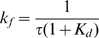 and 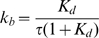.

An enzymatic reaction of substrate S with enzyme E to produce product P was simulated by a collection of two elementary processes: 1) enzyme E bound to substrate S to form the enzyme-substrate complex ES; and 2) the complex ES dissociated into enzyme E and product P. The chemical equation can be written as
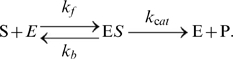
(3)The Michaelis-Menten formulation was avoided due to problems with the steady-state assumption [21],[43]. However, many papers and databases have provided only 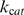 and the Michaelis constant 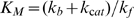 rather than 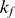 and 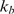. In such cases, it was assumed that 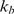 was four times larger than 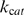 (i.e. 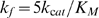 and 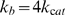), based on the default setting in GENESIS/Kinetikit simulator. (Tables S1, S2, S3).

### Intracellular signal transduction

Postsynaptic spines receive two presynaptic inputs: glutamatergic terminals from the cerebral cortex and dopaminergic terminals from the substantia nigra pars compacta. Plasticity of corticostriatal synaptic input results from phosphorylation of AMPA-type glutamatergic receptors, which promotes insertion into the postsynaptic membrane [20],[44],[45]. Below, the pathways linking glutamatergic and dopaminergic input to phosphorylation of AMPA receptors are delineated.

1. Glutamate release from the cortical presynaptic terminal increases calcium concentration in the postsynaptic spine via three mechanisms: i) calcium influx from NMDA- and AMPA-type glutamatergic receptor channels, ii) calcium efflux from the ER via 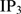 receptor channels following mGluR activation, iii) calcium influx from VGCC due to EPSPs of NMDA- and AMPA-type glutamate receptors, and back-propagation of action potentials when neural firing is evoked.
2. D1Rs binding to dopamine activates the olfactory-type guanine nucleotide-binding protein (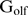), which then activates adenylyl cyclase 5 (AC5) by binding 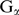 subunit of 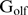. AC5 degrades ATP into cyclic adenosine 3′,5′-monophosphate (cAMP) [46] which then binds cAMP-dependent protein kinase (PKA), thereby disassociating the catalytic and regulation subunit. The catalytic subunit functions as an active PKA, which activates phosphodiesterase (PDE) and degrades cAMP, forming a negative feedback loop composed of cAMP, PKA, and PDE.
3. Calcium increases by glutamate and PKA activation by dopamine exhibit bi-directional interactions. 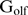 activation by D1R activates phospholipase C (PLC) in the mGluR pathway to induce calcium release from the ER. Calcium inhibits AC5 [47]–[49] and enhances degradation of cAMP by PDE via calmodulin (CaM).
4. CaM binding to calcium activates calcium-calmodulin-dependent protein kinase II (CaMKII), which is also activated by self-phosphorylation of Thr286 and de-phosphorylated by protein phosphatase 1 (PP1) [50]–[52]. CaMKII phosphorylates AMPA receptors, which promotes receptor insertion into the postsynaptic membrane.
5. In contrast, calcium activates protein phosphatase 2A (PP2A) [53], which is also activated by PKA phosphorylation. This mechanism involves several types of PP2A subunits, including catalytic C subunit, regulatory A subunit, and regulatory B subunit. Several subtypes of B subunit exist; one subunit binds calcium and another is phosphorylated by PKA [53],[54]. When these subunits bind the AC complex, PP2A functions as an enzyme and dephosphorylates AMPA receptors.
6. PKA also indirectly promotes phosphorylation of AMPA receptors by inhibiting PP1 via binding inhibitor 1 (I-1) and threonine 34 (Thr34) of DARPP32 [55]–[58] phosphorylated by PKA. PP1 activation can be regarded as the disinhibition resulting from release of these inhibitors. PP1 dephosphorylates AMPA receptors and CaMKII.
7. Calcium also activates protein phosphatase 2B (PP2B, or calcineurin) by binding calcium and CaM, and PP2B inhibits or activates AMPA receptors indirectly. PP2B dephosphorylates I-1 and DARPP32 at Thr34, both of which cause disinhibition of PP1, thereby inhibiting (triple-negative) AMPA receptors. PP2B also dephosphorylates and activates casein kinase 1 (CK1), which self-inhibits via autophosphorylation [59]. Subsequently, CK1 phosphorylates DARPP-32 at serine 137 (Ser137), which then suppresses the speed of PP2B-induced DARPP-32 dephosphorylation at Thr34 [55], [60]–[63], thereby facilitating (quadruple-negative) AMPA activity. Ser137 is dephosphorylated by PP2C [61], and DARPP-32 phosphorylation at Ser137 decreases the rate of dephosphorylation of phospho-Thr34 by PP2B [55]. CK1 also activates cyclin-dependent kinase 5 (Cdk5), a pathway that remains poorly understood. Therefore, the present study assumes the pathway to be one enzymatic reaction for simplicity. Cdk5 phosphorylates DARPP-32 at threonine 75 (Thr75) [64],[65].
8. Finally, there exists a positive (double-negative) feedback loop, composed of PKA, PP2A, and DARPP32 at Thr75: i) PKA activates PP2A [54], ii) PP2A dephosphorylates DARPP-32 at Thr75, and iii) DARPP-32 phosphorylated at Thr75 binds and inhibits PKA [66],[67]. PKA, PP2A, and DARPP-32 form a positive feedback loop, where PP2A activation disinhibits PKA [16]. Activation of this positive feedback loop can cause direct and indirect phosphorylation of AMPA receptors by PKA, as well as dephosphorylation by PP2A.

### Modeling strategy

The above-described signaling cascade, which links glutamatergic and dopaminergic inputs to AMPA receptor regulation, includes multiple excitatory and inhibitory pathways and feedback loops. This makes logical or intuitive inference of network behaviors virtually impossible; the outcomes depend on the strength and delay associated with each arrow in the diagram.However, logical or intuitive inference of network behaviors becomes virtually impossible, because the outcomes depend on strength and delay associated with each arrow in the diagram. This necessitates numerical simulation of a quantitative model of a signaling cascade to understand and prediction the dynamic behavior.

Therefore, the present study designed a kinetic model of the cascade with the concentrations of intracellular calcium and extracellular dopamine as the inputs and AMPA receptor concentration in the postsynaptic membrane as the output. However the cascade, which links glutamate stimulation to calcium response was not included in this model but will be addressed in a future study.

Similar to most large-scale cascade models, many reactions were adopted from previously published model [21],[22],[55],[66],[68],[69] or deposited the DOQCS database [42]. When available, models of striatal spiny neurons were utilized (e.g., DARPP-32, D1R, and AC5). Otherwise, Otherwise, hippocampal neuron models were adopted (e.g., CaM, CaMKII,PP2B, I-1, and AMPA receptor) by assuming that molecular processes are common between different brain areas. If no previous model was available (e.g., PP2A, PP1, CK1, and Cdk5), a reaction model was designed based on previous literature.

Because many of the reactions remain poorly understood, a number of assumptions and simplifications were necessary to design the cascade models. For instance, although DARPP32 contains at least four phosphorylation sites that affect its enzymatic properties, phosphorylation of Ser102 by CK2, which facilitates phosphorylation of Thr34 by PKA, was not modeled [70]. This was because the upstream regulation mechanisms for CK2 are now well known. Therefore, an 8-state model was designed for DARPP-32, with three phosphorylation sites: Thr34, Thr75, and Ser137.

CK1 activation is required for Cdk5 activation [64]. Although the cascade linking these two molecules has not yet been identified, a direct pathway from CK1 to Cdk5 has been hyphothesized [23]. Although reports have described PP1 phosphorylation by Cdk5 [71], a simple model was adopted from the DOQCS database, where only inhibition and disinhibition by I-1 and Thr34 were taken into account [72].

**Figure 3:**
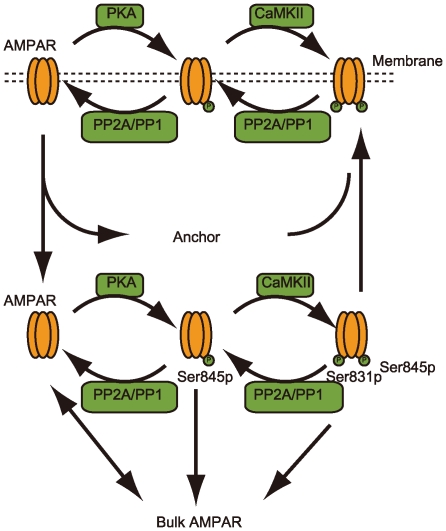
Schematic diagram of the AMPA receptor trafficking model. AMPA receptors are phosphorylated at Ser845 and Ser831 by PKA and CaMKII, respectively, and are also dephosphorylated by PP1 and PP2A. The phosphorylated AMPA receptors bind to anchor protein (Anchor) and are inserted into the cell membrane. In contrast, dephosphorylated AMPA receptors are removed from the membrane. AMPA receptors released from anchor protein are degraded and stored in cytosol (Bulk AMPAR).

AMPA receptor trafficking in the postsynaptic membrane was modeled using the state transition diagram shown in Fig. 3. AMPA receptors contain two phosphorylation sites - Ser845 phosphorylated by PKA and Ser831 phosphorylated by CaMKII. Therefore, a serial phosphorylation model was proposed for hippocampal neurons [73] where Ser831 was phosphorylated after Ser 845 phosphorylation.

Initially, the model was tested to determine whether it reproduced known features of calcium- and dopamine-dependent plasticity in medium spiny neurons. Subsequently, the dynamic characteristics of the model were analyzed to predict effects of experimental manipulation.

### Parameter setting and simulation

The entire model consisted of 72 reactions, with 132 reaction parameters. Among these, 83 parameters were retrieved from literature and model database. The remaining 49 parameters were hand-tuned to qualitatively reproduce the following properties:

1. The D1R agonist increases Thr34 phosphorylation levels [15], which was used to fit Thr34 responses to dopamine input.
2. Dopamine decreases Thr75 [16], which was used to fit the Thr75 response to dopamine input.
3. Group 1 mGluR agonist increases Thr75, Ser137, and Cdk5 activity [64], which was utilzed to fit Cdk5, Ser137, and Thr75 responses to 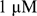 calcium input.
4. Glutamate decreases Thr75 [18], which was used to fit the Thr75 response to 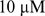 calcium input.
5. AMPA and NMDA decrease Thr34 and Thr75 [17],[74], which was employed to fit Thr75 and Thr34 responses to 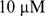 calcium input.
6. LTD induced by cortical high frequency stimulation leading to small increases in intracellular calcium is blocked by knocking out DARPP-32 [75]. This was used to fit synaptic efficacy by 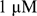 calcium input under normal and absence of DARPP-32 conditions.
7. LTP induced by cortical high frequency stimulation in Mg-free solution leading to large increases in intracellular calcium is blocked by knocking out DARPP-32 [75]. This was used to fit synaptic efficacy by 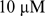 calcium input under normal and absence of DARPP-32 conditions.

Forms and parameters of all reactions are listed in Tables S1, S2, S3. Because many of the parameters affected multiple features of the model behavior, it was difficult to specify which parameter was responsible for the replication of each property.

Numerical simulations were implemented by GENESIS/kinetikit (http://www.genesis-sim.org/GENESIS/). It was assumed that the postsynaptic spine was a homogeneous volume of 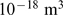 (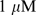 cubed), so that each molecular species concentration represented the state variables.

### Input time course

The two inputs to the cascade model comprised the concentrations of intracellular calcium, which were evoked by cortical glutamatergic input, and extracellular dopamine, which were evoked by nigral dopaminergic input. The time courses of the concentrations were approximated by the alpha function
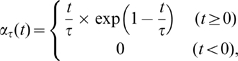
(4)which takes a maximum value of 1 when 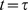.

**Figure 4:**
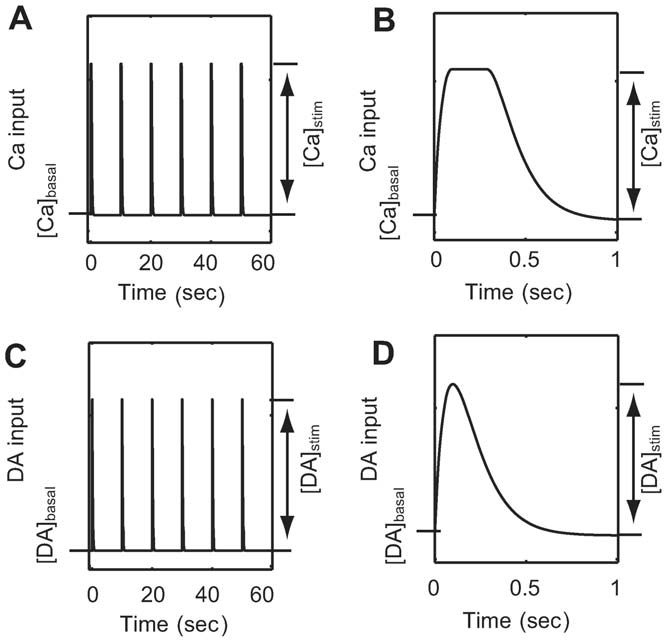
Transient time courses from two input sources. (A) Calcium input and (B) magnification from 0 to 1 second. (C) Dopamine input and (D) magnification from 0 to 1 second.

The intracellular calcium concentration induced by a train of 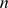 cortical spikes, which begin at time 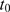 with 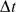 inter-spike interval (ISI), was simulated by
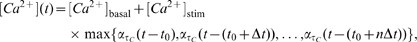
(5)where 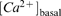 and 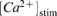 were the basal level and stimulus amplitude of calcium concentration, respectively (Fig. 4A). The maximum function, rather than temporal summation, of calcium transients was used to replicate calcium response data from D1R-expressing striatal neurons [76]. The time constant of the alpha function was 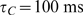 [77],[78]. 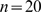 spikes at 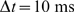 ISI (100 Hz) were simulated and repeated six times with 10-sec intervals (Fig. 4B). The concentrations used in the simulation were as follows: 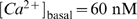 and 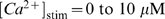.

The extracellular dopamine concentration, which was induced by a single presynaptic spike at time 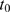, was simulated by:
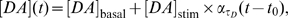
(6)where 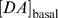 and 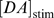 were the basal level and stimulus amplitude of dopamine concentration, respectively (Fig. 4C). The time constant of the alpha function was 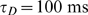 [78],[79]. Dopamine input simulation was repeated six times with 10-sec intervals (Fig. 4D). The concentrations used in the simulation were as follows: 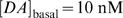 and 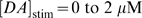.

## Results

First, the responses to intracellular molecules and AMPA receptor activation under four different levels of calcium and dopamine inputs were simulated. Then, changes in the post-synaptic AMPA receptors were predicted at different levels of calcium and dopamine inputs. Finally, *in silico* experiments with blockades of different pathways were developed to elucidate the dynamic mechanisms of calcium- and dopamine-dependent plasticity.

### Cascade responses to calcium and dopamine inputs

**Figure 5:**
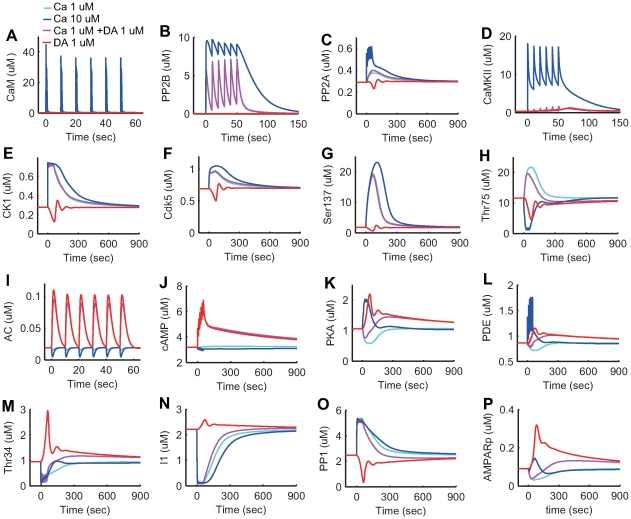
Transient activation responses of intracellular molecules from the original model. Line colors denote four different conditions: 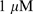 calcium influx without dopamine input (cyan); 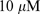 calcium influx without dopamine input (blue); 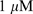 calcium influx coincident with 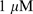 dopamine input (magenta); and 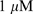 dopamine input in the absence of calcium influx (red). (A–O) Each plot indicates the activation state of each protein. (P) AMPARp indicates total concentration of phosphorylated AMPA receptor from at least one phosphorylation site.

The activities of intracellular molecules were simulated in response to four input conditions: i) weak calcium input alone (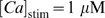 and 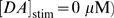); ii) strong calcium input alone (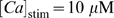 and 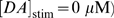); iii) dopamine input alone (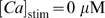 and 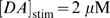). iv) weak calcium input coincident with dopamine input (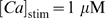 and 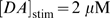); The detailed input forms are explained by Eqs. (4)–(6) in Materials and Methods, and the transient time courses are shown in Fig. 4. Results are shown in Fig. 5.

Direct downstream of calcium, CaM (Fig. 5A), PP2B (Fig. 5B), and PP2A (Fig. 5C) were moderately activated by weak calcium input (cyan), but more highly activated by strong calcium input (blue). In contrast, CaMKII (Fig. 5D), which self-phosphorylates, did not respond to weak calcium input (cyan), but responded drastically to strong calcium input (blue). The differential activation profiles of PP2A, which dephosphorylates AMPA receptors, and CaMKII, which phosphorylates AMPA receptors, can be a source of bi-directional plasticity due to calcium input.

CK1 (Fig. 5E) was activated by PP2B, but the response to strong calcium input was saturated due to a self-inhibitory mechanism. CK1 subsequently activated Cdk5 (Fig. 5F) and the Ser137 phosphorylation site of DARPP-32 (Fig. 5G).

Phosphorylation of Thr75 in DARPP-32 (Fig. 5H) increased with weak calcium input (cyan) via Cdk5 activation (Fig. 5), but decreased with strong calcium input (blue) via PP2A activation (Fig. 5C). This bi-directional calcium effect on Thr75 was consistent with experiments showing phosphorylation of Thr75 with a glutamate receptor agonist [17],[18].

Downstream of the D1Rs, AC5 (Fig. 5I) increased with dopamine input, but decreased with strong calcium input due to calcium inhibition. cAMP concentration (Fig. 5J) increased or decreased depending on AC5 activation level, and subsequently slowly decayed. Phosphorylated PKA (Fig. 5K) decreased with weak calcium input (cyan) and increased with strong calcium input (blue), mirroring the bi-directional changes of Thr75 (Fig. 5H). PKA increased at a slower rate with dopamine input (red), subsequent to increased cAMP. Simultaneous stimulation of weak calcium and dopamine resulted in a bi-phasic response, including an initial dip followed by a sustained elevation. PDE activation (Fig. 5L) was similar to the activation profile of PKA.

Dopamine input (red) resulted in increased Thr34 phosphorylation of DARPP-32 (Fig. 5M) via PKA activation. Calcium input (cyan and blue) reduced Thr34 phosphorylation due to stronger inhibition by PP2B. The decreased Thr34 phosphorylation due to calcium input was consistent with experimental results utilizing AMPA and NMDA [17]. Coincident calcium input (magenta) reduced the response of Thr34 to dopamine input (red). These results were consistent with experimental responses to different levels of dopamine and NMDA inputs [74].

Dopamine input alone increased phosphorylation of Inhibitor-1 (I-1) (Fig. 5N) via PKA activation. However, I-1 phosphorylation decreased due to either weak or strong calcium input, or simultaneous calcium and dopamine inputs, via PP2B inhibition. Phosphorylation of PP1 (Fig. 5O) was opposite to that of I-1 by dopamine input (red), but similarly phosphorylated by both strong (blue) and weak (cyan) calcium inputs, even under simultaneous dopamine input (magenta).

Finally, via phosphorylation by CaMKII (Fig. 5D) and PKA (Fig. 5K), and dephosphorylation by PP2A (Fig. 5C) and PP1 (Fig. 5O), AMPA receptor phosphorylation at Ser845 decreased due to weak calcium input, but increased due to strong calcium input and simultaneous calcium and dopamine inputs (Fig. 5P).

### Dopamine- and calcium-dependent synaptic plasticity

**Figure 6:**
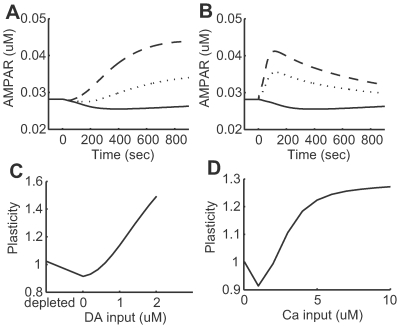
Dopamine- and calcium-dependent synaptic plasticity reproduced by the model. (A) Transient time courses of synaptic efficacy induced by 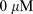 (solid line), 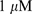 (dotted line), and 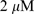 (dashed line) dopamine input coincident with 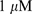 calcium input. (B) Transient time courses of synaptic efficacy induced by 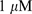 (solid line), 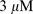 (dotted line), and 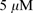 (dashed line) calcium input without dopamine input. In all cases from (A) and (B), input was initiated at 0 seconds and synaptic efficacy was evaluated by the number of AMPA receptors in the post-synaptic membrane. (C) Synaptic plasticity as a function of dopamine input with 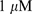 calcium input. The dopamine concentration was fixed at 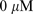 in the depleted condition, but set to 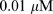 steady state in the remaining conditions. (D) Synaptic plasticity as a function of calcium input. For (C) and (D), plasticity was evaluated by the ratio of the number of AMPA receptors in the post-synaptic membrane prior to and 10 minutes after stimulation onset.

Fig. 6A shows the time course of synaptic efficacy (AMPA receptor concentration in the post-synaptic membrane) induced by different levels of dopamine input coincident with a weak calcium input. While the absence of dopamine input caused depression of the synapse (solid), increased dopamine levels resulted in potentiation.

Fig. 6B shows the time course of synaptic efficacy in three different levels of calcium input without dopamine input. While weak calcium input causes depression, increased calcium input resulted in potentiation.

Synaptic efficacy was evaluated 10 min after conditioning as an index of long-term synaptic plasticity. Synaptic efficacy increased with increasing dopamine input coincident with calcium input (Fig. 6). In conditions of dopamine depletion, where both 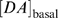 and 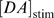 were set at 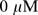, the calcium input did not alter synaptic efficacy. These results were in accordance with dopamine-dependent synaptic plasticity [27], as characterized in Fig. 1A.

Fig. 6D shows synaptic plasticity dependence on calcium input levels in the absence of dopamine input. Weaker calcium input resulted in LTD, but stronger calcium input caused LTP. These results were consistent with previous experimental observations [11],[28],[29], as schematized in Fig. 1B.

To further clarify the interactions between calcium and dopamine inputs and the roles of molecules in the signaling cascade, 2D maps of synaptic plasticity were plotted with different levels of calcium and dopamine inputs using standard and modified models.

**Figure 7:**
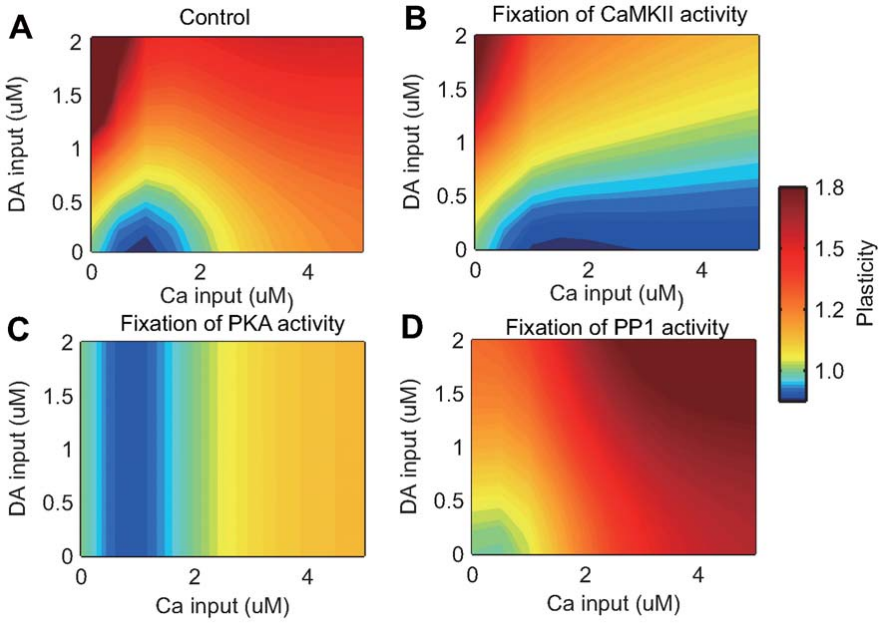
Contour plot of synaptic plasticity during dopamine and calcium input. Panels (A–D) show results from four different conditions: (A) control with the original model; (B) fixation of CaMKII activity; (C) fixation of PKA activity; and (D) fixation of PP1 activity. The quantitative evaluation of synaptic plasticity was identical to Fig. 4. Green (corresponding to 1.0 in the right color-bar) indicates areas where synaptic efficacy was not altered. Hotter and colder colors indicate areas where LTP and LTD are induced, respectively.

Fig. 7A shows synaptic plasticity after 10 minutes stimulation in the standard model. LTD was induced by weak calcium input in the absence of dopamine (blue area), and LTP was induced by either strong calcium or strong dopamine input (red area).

When CaMKII activation was fixed at a steady-state level (Fig. 7B), increased calcium input did not induce LTP. Rather, LTD occurred only at low levels of dopamine input. When PKA was fixed at the steady-state level (Fig. 7C), dopamine-dependent plasticity disappeared. Fixing PP1 produced LTP, regardless of the strength of calcium and dopamine inputs (Fig. 7D). The potentiation induced by strong dopamine alone disappeared, because the disinhibition due to decreased PP1 (corresponding to the red line in Fig. 5O) was removed.

### Dynamic mechanisms behind calcium and dopamine-dependent plasticity

#### The role of the CK1-Cdk5 pathway in calcium-dependent plasticity

**Figure 8:**
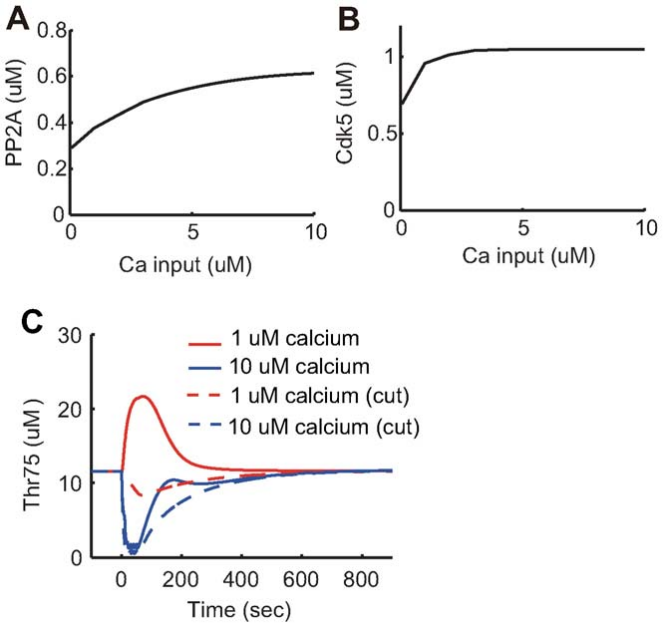
The role of the CK1-Cdk5 pathway. The maximum response of (A) Cdk5 and (B) PP2A activities to different levels of calcium input. (C) Altered transient responses of phosphorylated Thr75 by removing the Ck1-Cdk5 pathway. The solid lines are responses from the original model. Dotted lines are the responses from the modified model, where the CK1-Cdk5 pathway was removed from the original model. Different levels of calcium input are denoted by different colors: red for 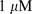 calcium input; and blue for 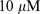 calcium input.

To determine the mechanisms of bidirectional change in Thr75 phosphorylation due to weak and strong calcium inputs (Fig. 5H), Cdk5 and PP2A responses to different levels of calcium inputs were analyzed (Fig. 8). Although Cdk5 was steeply activated at a low level of calcium input (Fig. 8A), PP2A was gradually activated with increased calcium input levels (Fig. 8B). The Cdk5 effect was dominant with a weak calcium input, leading to Thr75 phosphorylation (cyan line in Fig. 5H). When Cdk5 was saturated due to stronger calcium input, the stronger effect of PP2A resulted in Thr75 dephosphorylation (blue line in Fig. 5H). To verify the role of the CK1-Cdk5 pathway, a simulation was performed with the removal of this pathway. Results demonstrated that inhibition of the CK1-Cdk5 pathway drastically decreased Thr75 phosphorylation under conditions of weak calcium input (red lines in Fig. 8C).

#### The role of DARPP-32

Although DARPP-32 affects striatal synaptic plasticity, the signaling cascade diagram (Fig. 2) includes pathways to PKA and PP1 from either dopamine input or calcium input without going through DARPP-32.

**Figure 9:**
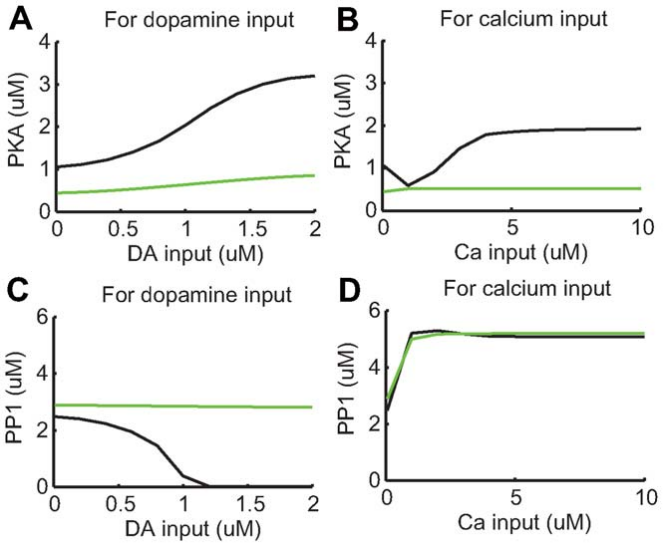
Responses of PKA and PP1 in the absence of DARPP-32. (A–B) Maximal responses of active PKA to various levels of dopamine and calcium input, respectively. (C–D) Maximal responses of active PP1 to various levels of dopamine and calcium input, respectively. For all panels, black lines indicate results from the original model (control), and green lines indicate results from the modified model, where DARPP-32 is fixed at 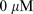 (DARPP-32 knockout condition).

To examine the role of DARPP-32 in synaptic plasticity, a DARPP-32 knockout was simulated by maintaining a DARPP-32 concentration of 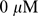 (Fig. 9). Under this condition, dopamine-induced PKA activation became much weaker (Fig. 9A) and stayed almost constant with increased calcium input (Fig. 9B). Dopamine-induced PP1 inhibition was abolished (Fig. 9C), while its activation by calcium input was maintained (Fig. 9D).

**Figure 10:**
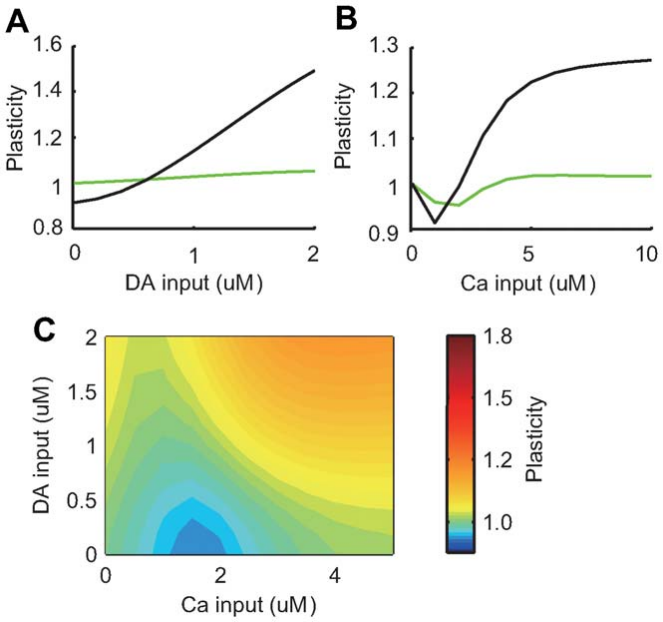
Synaptic plasticity in the absence of DARPP-32. (A) Synaptic plasticity due to varying strengths of dopamine input combined with 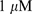 calcium input. (B) Synaptic plasticity due to varying strengths of calcium input without dopamine input. Black lines indicate results from the original model (control), and green lines indicate results from the modified model, where DARPP-32 is fixed at 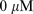 (DARPP-32 knockout condition). (C) Contour plot of synaptic plasticity in the DARPP-32 knockout condition as a function of calcium and dopamine input.

Fig. 10 shows dopamine- and calcium-dependent plasticity in the absence of DARPP-32. Dopamine-dependent plasticity almost disappeared, and calcium-dependent plasticity lost the LTP component and retained only a weak LTD component. These results were consistent with experimental results from DARPP-32 knockout mice [75].

These results suggested: 1) PKA was critical for both dopamine- and calcium-dependent LTP; 2) PP1 played an important role in calcium-dependent LTD; and 3) DARPP-32 played a critical role in the bi-directional regulation of dopamine- and calcium-dependent synaptic plasticity.

#### PKA-PP2A-DARPP-32 positive feedback loop

Results from DARPP-32 knockout simulation suggested that direct activation of PKA through the D1R-AC5-cAMP pathway was not sufficient. In addition, amplification via the PKA-PP2A-Thr75-PKA-positive (double-inhibitory) feedback loop played an essential role in LTP induction.

**Figure 11:**
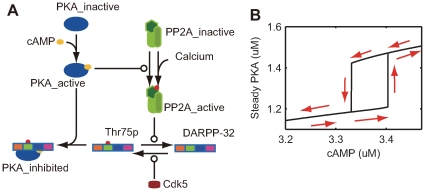
Hysteresis of PKA-PP2A-DARPP-32 positive feedback loop. (A) Schematic diagram of the sub-network forming the PKA-PP2A-DARPP-32 positive feedback loop. Blocks indicate different molecular states. Specifically, DARPP-32 has four phosphorylation cites (Thr34, Thr75, Ser102, and Ser137), which are indicated by different colors in this diagram. Round arrowheads are enzymatic actions and red dots indicate phosphorylated states. (B) Active PKA changes at steady states, with gradual changes in cAMP concentration at fixed concentrations of calcium at 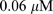 and Cdk5 at 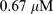. First, cAMP concentration was set to 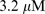, and active PKA steady state was calculated by COPASI. Subsequently, cAMP concentration was increased by a step of 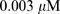 to 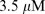, and steady state level of active PKA was calculated at each setting. Next, cAMP concentration was reduced by a step of 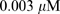 to 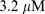, and steady state of active PKA was analyzed again. The arrows along the lines show the direction of the trajectory in the two-dimensional space of cAMP conditions and steady states of active PKA.

To determine the effects of this positive feedback loop, the sub-network dynamics were analyzed, which consisted of a PKA-PP2A-Thr75 loop containing calcium, Cdk5, and cAMP as parametric inputs (Fig. 11A). Calcium and Cdk5 were set to baseline levels, and cAMP concentration was gradually increased starting with 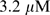 (Fig. 11B). The steady-state level of active PKA gradually increased to approximately 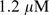, but drastically increased to approximately 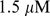 when the cAMP concentration exceeded 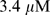. In contrast, when the cAMP concentration was gradually decreased from 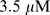, steady-state PKA activation gradually decreased to approximately 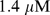 and then was abruptly reduced to around 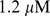 as cAMP concentrations fell below 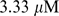 (see Fig. 11 caption for an exact description). This hysteresis suggested bi-stability of the positive feedback loop at intermediate levels of cAMP input.

**Figure 12:**
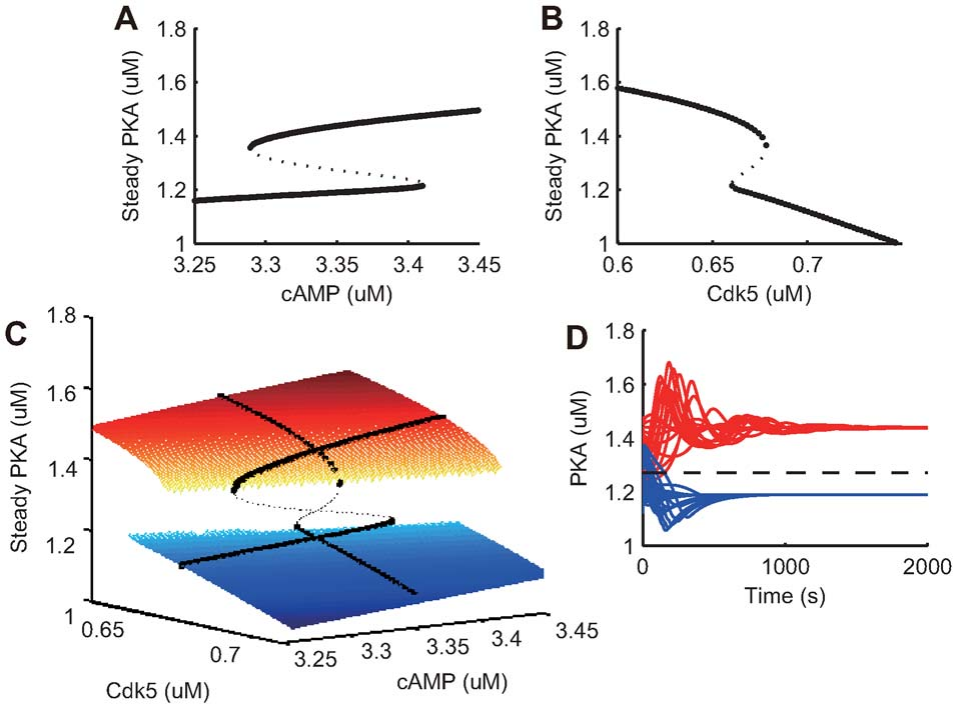
Bi-stability of PKA-PP2A-Thr75 positive feedback. (A, B) Bifurcation diagrams created by identification of steady states using the Newton method and determination of stabilities using the eigenvalues of the Jacobian. Large points indicate stable steady states and small points indicate unstable steady states. (A) Bifurcation diagram for the altered cAMP, with fixed parameters of 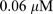 calcium and 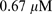 Cdk5. The subsystem has one stable state when cAMP is less than 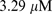 or greater than 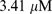. At middle range of cAMP, three steady states exist: two stable states and one unstable state. (B) Bifurcation diagram for the altered Cdk5, with fixed parameters of 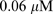 calcium and 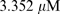 cAMP. The subsystem has one stable state when Cdk5 is less than 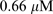 or greater than 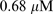. At middle range of Cdk5, three steady states exist: two stable states and one unstable state. (C) Steady state level of PKA in the 2D parameter space of cAMP and Cdk5. The calcium concentration was fixed at 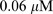. The blue and red planes are steady states of PKA at low and high levels, respectively. The black dots indicate steady states with Cdk5 fixed at 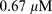 or cAMP fixed at 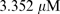, as plotted in panels A and B. (D) PKA trajectories from several initial conditions at a cAMP level of 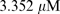 and Cdk5 level of 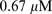. The trajectories funnel toward a stable steady state. The dotted line indicates PKA levels at an unstable steady state.

To more rigorously examine subnetwork bistability (Fig. 11A), a steady state analysis was performed with COPASI [80]. For each cAMP and Cdk5 level, steady states were identified from multiple initial states using the Newton method, and stabilities were determined by calculating eigenvalues of the Jacobians. Fig. 12A shows the resulting bifurcation diagrams using cAMP level as the parameter. The subsystem exhibited one stable state when cAMP was less than 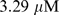 or greater than 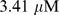. In the cAMP middle range, the subsystem exhibited three steady states: two asymptotically stable steady states and one unstable steady state in the middle. Bistability was also observed in an analysis using Cdk5 level as the parameter (Fig. 12B).

Fig. 12C shows the bifurcation diagram of the two-dimensional parameter space by cAMP and Cdk5 levels. Two planes of stable steady states (blue and red) existed, which were connected by a band of unstable stable states (not shown for clarity). The higher Cdk5 level shifted the threshold of cAMP input for PKA activation (the end of blue plane) higher. As Cdk5 is activated by the calcium input and cAMP is activated by the dopamine input, this interdependency of Cdk5 and cAMP for PKA activation could be a cause of calcium and dopamine interaction in producing LTP and LTD. In fact, this bifurcation diagram is consistent with our analysis of the plasticity in the 2D parameter space of calcium and dopamine inputs shown in (Fig. 7B), where the activation of AMPA receptors by CaMKII was held constant. In addition, PKA trajectories from several initial conditions were confirmed and plotted in Fig. 12D. The trajectories converged toward one of the two stable steady states.

**Figure 13:**
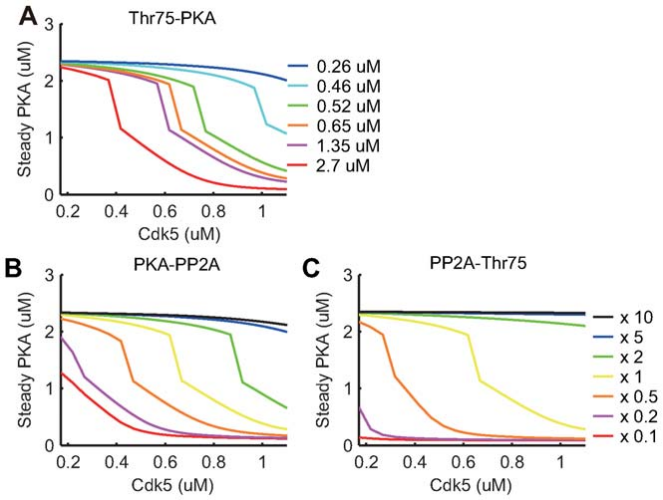
Robustness of the PKA-PP2A-DARPP-32 positive feedback loop. (A–C) Robustness of the threshold-like PKA activation as a function of total concentration of Cdk5 in the sub-system shown in Fig. 9, when three parameters were independently altered: (A) A dissociation constant Kd in a reaction where Thr75 is dissociated from inhibited PKA, was given by 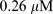 (blue), 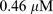 (cyan), 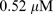 (green), 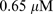 (orange), 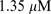 (magenta) or 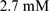 (red); (B) A catalytic constant 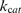 in a reaction where active PKA phosphorylates PP2A, is given by 10 times (black), 5 times (blue), 2 times (green), control (yellow), 0.5 times (orange), 0.2 times (magenta), 0.1 times (red), larger than the control value in the original model (yellow); and (C) A catalytic constant 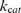 in a reaction where active PP2A dephosphorylates Thr75, is given by 10 times (black), 5 times (blue), 2 times (green), control (yellow), 0.5 times (orange), 0.2 times (magenta), 0.1 times (red), larger than the control value in the original model (yellow). Please note that the dissociation constant Kd in panel (A) was set at 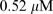 in our original model while it was said to be 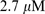 in an experimental study [57].

To test the robustness of the threshold dynamic of the positive feedback loop, the dissociation constants in the loop were varied up to ten-fold. The stationary concentration of active PKA, which started at a low initial level (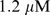), was observed with different level of Cdk5. As shown in Fig. 13, active steady state PKA abruptly decreased (failed to increase to the upper branch) when initial Cdk5 concentrations exceeded a threshold. Although the threshold value of Cdk5 varied according to altered dissociation constants, the threshold behavior was robust under wide ranges of model parameters. These results suggested that bistable dynamics of the positive feedback loop by PKA, PP2A, and Thr75 of DARPP-32 contributed to a robust nonlinear threshold response of PKA.

#### Baseline dopamine level

**Figure 14:**
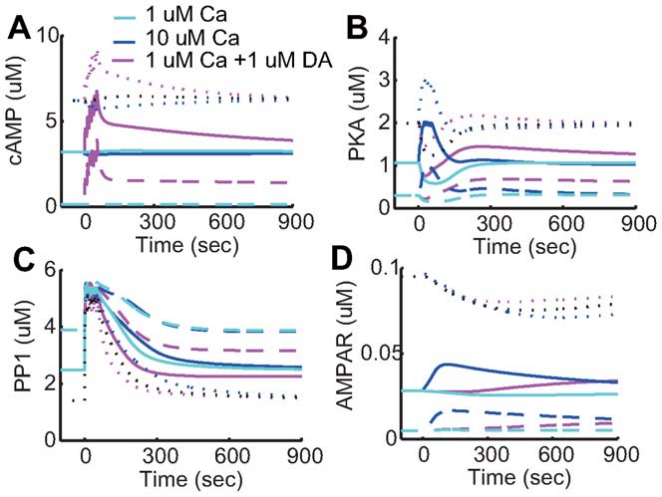
Transient responses at high basal dopamine levels. Time courses of (A) cAMP, (B) PKA, (C) PP1 and (D) AMPA receptor in the post-synaptic membrane, respectively, when basal dopamine level was altered. The cyan lines indicate 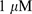 calcium influx, the blue lines indicate 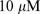 calcium influx without dopamine input, and the magenta lines indicate 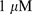 calcium influx together with 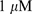 dopamine input. The solid lines indicate the 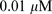 basal dopamine (control) condition, the dotted lines indicate the 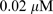 condition, and the dashed lines indicate the 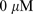 basal dopamine (dopamine depletion) condition.

It was analyzed how striatal plasticity is affected by the baseline concentration of dopamine, which is known to be altered in Parkinson's disease and drug addiction (Fig. 14). At increased basal levels of dopamine (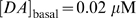; dotted lines), the steady-state levels of active PKA and phosphorylated AMPA receptor were two times greater than control levels (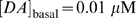; solid lines). From this high-level initial state, even strong calcium input, as well as simultaneous calcium and dopamine inputs, resulted in LTD. Because initial levels of PP1 decreased (Fig. 14C) with increasing PKA inhibition (Fig. 14B) and responded in a larger amplitude to both calcium and dopamine inputs, causing dephosphorylation of AMPA receptors. Under dopamine depletion conditions (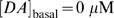; dashed lines), the steady-state level of the phosphorylated AMPA receptor was less than the control (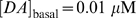). Because of a decreased active PKA steady state, active PP1 level was initially higher with decreased responses to both calcium and dopamine inputs. This resulted in LTP with calcium input, as well as simultaneous calcium and dopamine inputs (Fig. 14D, dashed lines).

#### Timing of calcium and dopamine inputs

The effect of calcium and dopamine input timing was tested under three conditions: 1) simultaneous initiation of calcium and dopamine inputs (control); 2) dopamine input preceding calcium input by 500 ms (DA preceding condition); and 3) dopamine input following calcium input with a 500-ms delay (DA following condition). Differences in input timing affected the AC5 response, with the DA following condition resulting in the greatest amplification(Fig. S1A). The amplified AC5 response was propagated through cAMP (Fig. S1C) to PKA (Fig. S1D). As a consequence, LTP was most effectively induced when dopamine input followed calcium input (Fig. S1E).

## Discussion

To understand cortico-striatal synaptic plasticity at the molecular level, a signaling cascade model for a single spine of a striatal medium spiny neuron was constructed. Strong calcium influx induced PKA activation through the PP2A-DARPP-32 pathway, which resulted in LTP. PKA was normally inhibited by DARPP-32 phosphorylation at Thr75. In addition, weak calcium input resulted in DARPP-32 phosphorylation at Thr75 through the PP2B-CK1-Cdk5 pathway, which ultimately resulted in PKA inhibition and LTD. In contrast, a strong calcium influx resulted in PP2A dephosphorylation at Thr75, ultimately disinhibiting (activating) PKA and leading to LTP. Previous studies have shown that CaMKII, not PKA activation, causes hippocampal LTP [81]. However, a marked feature of the striatal synaptic plasticity is that both CaMKII and PKA activation contribute to calcium-dependent LTP.

Dopamine activatd PKA through the AC5-cAMP pathway and then PKA activity was amplified by a PKA-PP2A-DARPP-32 positive feedback loop, leading to a threshold phenomenon. PKA phosphorylated AMPA receptors directly and indirectly reduced dephosphorylation of AMPA receptor by PP1 through DARPP-32 on Thr34 and I-1. These dual pathways provided for a robust regulation of AMPA receptor by PKA.

The major findings of this study are discussed below in relation to previous modeling and experimental studies.

### Comparison with previous models

Several studies have modeled signal transduction in medium spiny neurons [21]–[23]. The novelty of the present model is the incorporation of AMPA receptor phosphorylation and membrane trafficking to directly assess the effects of cascade dynamics on striatal synaptic plasticity. This allowed for the reproduction of both LTD and LTP in calcium- and dopamine-dependent plasticity and to predict interactions between calcium and dopamine inputs, as shown in Fig. 7, and effects of various manipulations on striatal synaptic plasticity. Embedding of the present model in a complete neuronal model, or even a neural network model, enables the assessment of the role of calcium- and dopamine-dependent plasticity in cellular and network functions. The model can also serve as the basis for building simplified signaling cascade models for large-scale simulation and theoretical analysis.

The present signaling cascade model involving DARPP-32 differs from previous models in several points. The factors incorporated by this model but not by existing models [21]–[23] were inhibition of PDE by PKA, Ser137 effect on Thr34, and inhibition of PP1 by I-1. The CK1-Cdk5 pathway, which has been previously described [23], was critical for reproducing bidirectional phosphorylation of Thr75, which was dependent on calcium input intensity. In addition, the present study performed a rigorous analysis of bistability of positive feedback loop formed by PKA, PP2A, and DARPP-32 on Thr75, which was a source of a threshold-like response of PKA activity to both dopamine and calcium inputs.

The model prediction of Thr34 and Thr75 responses to dopamine and calcium input were consistent with the Fernandez model [21] if the calcium input levels from the Fernandez model were regarded as the strong calcium input for the present model. However, simultaneous calcium and dopamine inputs resulted in Thr34 dephosphorylation in the present model, but phosphorylation in the Lindskog model [22]. This discrepancy could be due to inactivation by the calcium-PP2B-Thr34 pathway was stronger than activation by the PKA-Thr34 pathway in present model.

### CK1-Cdk5 pathway

DARPP-32 phosphorylation on Thr75 has been shown to because of glutamate, AMPA, or NMDA exposure, but returns to normal level within 10 min [17],[18]. In addition, an mGluR agonist has been shown to potentiate Cdk5 activation and phosphorylation of DARPP-32 on Thr75 and Ser137, and returns to baseline levels after peaking at 2 min [64]. Assuming that an mGluR agonist induced weak calcium levels, and glutamate, AMPA, or NMDA produced strong calcium input, those experimental results were consistent with the present results, as shown in Fig. 5H.

In present model, phosphorylation of DARPP-32 on Thr75, as a result of weak calcium input, takes place through the CK1-Cdk5 pathway. Although CK1 activation is required for Cdk5 activation through signaling from mGluR [64], it is not known whether the pathway from CK1 to Cdk5 is direct. Similar to a previous model, the present study assumed direct activation of Cdk5 by CK1 for simplicity [23]. Alternative mechanisms for inhibition of PP2A dephosphorylation on Thr75 exist - either through the calcium-AC5-cAMP-PKA pathway or the calcium-CaM-PDE-cAMP-PKA pathways. More quantitative data on the strengths of these pathways and additional *in silico* experiments are necessary to definitely determine the role of the CK1-Cdk5 pathway in calcium-dependent LTD.

### AMPA receptors

AMPA receptor trafficking in the present model was derived from Hayer's model [82]. The primary modification comprised sequential phosphorylation of Ser845 by PKA followed by Ser831 phosphorylation by CaMKII, as proposed by Lee *et. al.* [83]. However, the LTP mechanism in the present striatal model differed from the hippocampal LTP by Lee *et. al.* [83]. Previous results demonstrated that the phosphorylation of Ser845 did not increase during LTP [83], and the present model showed that the phosphorylation of Ser845 increased during dopamine-dependent LTP, but did not increase during calcium-dependent LTP. In addition, PKA was required for striatal LTP [75] To address this feature in the present striatal model, most of the AMPA receptors were dephosphorylated at the baseline. This prediction was consistent with the lower phosphorylation level of Ser845 by reduced PKA levels due to inhibition by DARPP-32 in the striatum [69].

It should be noted, however, that the observation of sequential AMPA receptor phosphorylation by Lee *et al.* [83] in the hippocampus did not exclude a parallel phosphorylation model. It could be interpreted as a result of high PKA and low PPI concentration at the baseline in the hippocampus. It is a subject of future study whether a parallel phosphorylation model can also reproduce the striatal synaptic plasticity.

D1-type neurons express GluR1 and GluR2/3 in the spines [84],[85]. A previous hippocampal study [86] showed that GluR1 subunit trafficking was a result of stimulation, but that GluR2 subunit trafficking was constitutive. In addition, chronic treatment with the antidepressant maprotiline increases GluR1, but not GluR2 [87]. Moreover, GluR2-lacking AMPA receptors exhibit larger single-channel currents than GluR2-expressing AMPA receptors [88]. For these reasons, trafficking of GluR1, but not GluR2, was modeled in the present study to ascertain whether synaptic plasticity responded to stimulus.

Some theoretical studies [89],[90] have predicted that NMDA receptor-mediated calcium influx results in bidirectional synaptic change. However, these studies modeled only AMPA receptor phosphorylation, but not trafficking, and also did not consider striatal synaptic plasticity.

Although the present model considered the number of AMPA receptors in the postsynaptic membrane as a measure of synaptic efficacy, previous studies have suggested that the conductance of AMPA receptor varies according to the phosphorylation state. For example, Ser831 phosphorylation increases conductance [91] and Ser845 phosphorylation increases open probability [92],[93]. If these effects are taken into consideration, the amplitude of LTP could be larger, as observed in experiments [8]–[11].

### Bistability and long-term plasticity

Threshold dynamics due to the bistability of the positive feedback loop of PKA, PP2A, and Thr75 on DARPP-32 played an important role in reverting the LTD to LTP in dopamine-dependent plasticity. However, when embedded into the entire system, the loop did not exhibit complete bistability, as demonstrated by gradual conversion of synaptic conductance to baseline levels (Fig. S2). The possible mechanisms for longer-lasting synaptic plasticity are described below.

First, bistability of some proteins in the cascade has been reported, such as the bistability of CaMKII phosphorylation [82]. However, CaMKII activity did not last for an extended period of time in the present model. This was consistent with a previous study [94], which reported that CaMKII activity returns to baseline within 2–5 min. Hayer *et. al.* observed bistability of AMPA receptor phosphorylation and Catellani *et. al.* [73] mathematically determined bistability in the sequential AMPA receptor phosphorylation model. These bistable mechanisms were not incorporated in the present model, but may contribute to synaptic changes over longer periods of time.

Second, the present model did not consider increased levels of AMPA receptors and other proteins as a result of gene transcription. A possible link from the current model to longer-term synaptic plasticity is cAMP-response element binding protein (CREB), which controls gene transcription for longer-term synaptic plasticity in the striatum [95]. CaMKII, PKA, and PP1 directly activate CREB, but also indirectly via extracellular signal-regulated kinase (ERK), which activates CREB [35],[96]. In addition, calcium activates mitogen-activated protein kinase kinase (MEK), which activates ERK [97].

PP1 activates striatal enriched phosphatase (STEP) [98], which inhibits ERK, and PKA inactivates STEP. As a result, CREB is inhibited by PP1 and activated by CaMKII and PKA. Therefore, activation of CaMKII and PKA, as well as inhibition of PP1, which results in AMPA receptor phosphorylation, can also trigger gene transcription through CREB activation.

### Model robustness

Approximately half of the model parameters were based on previous reports and databases [21],[22],[42],[55],[66],[68],[69], and the remaining half were designed to reproduce experimental findings [15]–[18],[39],[46],[64],[74]. Model behavior robustness was determined by altering the kinetic parameters of the PKA-PP2A-Thr75 loop up to ten-fold (Fig. 13). Persistence of nonlinear threshold behavior, despite shifts in thresholds, was also verified. Although the present model parameters reflected some uncertainty, the model served as a useful starting point for exploring the mechanisms influencing corticostriatal synaptic plasticity by testing alternative parameter values or incorporating additional pathways. The present model did not include a number of known pathways such as the effect of DARPP-32 Ser102 on phosphorylation of Thr75 [99].

### Different types of corticostriatal synaptic plasticity

Membrane potential of striatal medium spiny neurons shifts between up- and down-states, depending on cortical inputs [100]. During the up-state, LTP is induced by cortical stimuli, even without dopamine input [31]–[34]. LTP is also induced by cortical stimulation in a magnesium-free solution [11],[28],[29]. Both cases reflect calcium-dependent plasticity because of the large calcium influx through NMDA receptors.

Two types of medium spiny neurons exist: D1 receptor-expressing neurons that project to the direct pathway, and D2 receptor-expressing neurons that project to the indirect pathway [101],[102]. In D1 neurons, dopamine increases cAMP via G-proteins and AC5, similar to the present model. However, in D2 neurons dopamine inhibits AC5 and decreases cAMP so the effect of dopamine input is opposite to that in D1 neurons.

### Striatal synaptic plasticity and reinforcement learning

Schultz *et. al.* recorded the activities of dopamine neurons in the substantia nigra in monkeys and found that dopamine neurons encode error signals of reward prediction [103].

The reinforcement learning model of the basal ganglia posits that striatal neurons learn to compute expected reward based on the reward prediction error signal carried by dopamine neuron firing [103]. Dopamine-dependent synaptic plasticity plays a major role in learning. The medium spiny neurons are depolarized by glutamatergic inputs from the cortex that represent a sensory or a contextual state. When the acquired reward is more than expected, phasic dopamine neuron firing would induce LTP of the activated cortico-striatal synapses. On the other hand, if the reward is less than expected, a pause in dopamine neuron firing would cause LTD of those synapses. The glutamatergic input would not only cause depolarization and firing, but also induce changes in molecular states, such as the phosphorylation level of DARPP-32 and/or shift the threshold of the positive feedback loop, which would serve as the short-term memory of preceding states.

To support this scenario, the temporal order of calcium and dopamine input is a critical factor. Assuming that calcium flux by glutamatergic input is a fast process, the synaptic efficacy should be potentiated when calcium input (associated with a sensory or contextual state) precedes dopamine input (associated with a reward prediction error signal). Our model is consistent with this point (Fig. S1). On the other hand, our model also predicts that the effect of the temporal order on synaptic plasticity is not strong enough. This suggests additional interactions between dopamine and calcium signaling. For example, dopamine facilitates L-type calcium channels, which affect the calcium influx through the interaction of glutamate receptor activation and and back-propagating action potentials. To more precisely simulate calcium dynamics, we have to construct a whole neuron model [104] and combine it with the signaling cascade model.

### Dopamine-calcium interaction

There are several interaction pathways between calcium and dopamine signaling. In the upstream of PKA, calcium directly inhibits AC5 and indirectly cAMP through CaM and PDE. While calcium inhibition of AC5 depended on the timing between calcium and dopamine, PDE inhibition of cAMP did not depend on this timing very much. The stronger interaction of dopamine and calcium on PKA was through DARPP-32. Weak calcium input inhibited PKA through the phosphorylation of Thr75 by Cdk5, but strong calcium input activated PKA through the dephosphorylation of Thr75 by PP2A. While dopamine input reduced the increase of Thr75 by a weak calcium input, it did not affect the decrease of Thr75 by a strong calcium input.

Furthermore, the subsystem around the PKA-PP2A-DARPP-32 positive feedback loop showed bistability while PKA activity showed a threshold like response to cAMP activation by dopamine input. However, this loop became mono-stable with both activation of Cdk5 by a weak calcium input, leading to a low level of PKA, and by activation of PP2A by a strong calcium input, leading to a high level of PKA.

### Drugs and DARPP-32

Addictive drugs (e.g. cocaine and amphetamine) increase the basal level of dopamine by inhibiting the reuptake of dopamine and facilitating the release of presynaptic dopamine [5]. They ultimately decrease DARPP-32 phosphorylation on Thr75 and increase it on Thr34 [105]. In our model, increased basal dopamine levels caused LTD with the calcium and dopamine inputs which caused LTP under control conditions (Fig. 14). This result is consistent with the theory that the value of everything except for drugs decreases because of the impairment of appropriate learning in drug addiction [106].

## Supporting Information

Figure S1 Transient responses to different temporal orders of calcium and dopamine inputs. (A–D) Time courses of AC, PDE cAMP, and PKA, respectively, in responses to 10 µM calcium and 1 µM dopamine input. Line colors denote four different temporal orders: dopamine followed by calcium with a 500 ms delay (red line); dopamine together with calcium (blue line); dopamine preceding calcium with a 500 ms delay (green line); (E) Timing-dependent plasticity when 1 µM dopamine input and 1 µM calcium input are given. The timing-effect on synaptic efficacy, denoted by the horizontal axis, was evaluated as the fraction of the synaptic efficacy in each timing condition over that in the condition where dopamine and calcium inputs were given simultaneously.
(0.69 MB EPS)

Figure S2 Synaptic plasticity at 40 min. (A) Time course of AMPA receptors. The black line indicates 1 µM calcium input. The red dotted and dashed lines indicate 3 µM and 5 µM calcium input, respectively. The green dotted and dashed lines indicate 1 µM and 2 µM dopamine input combined with 1 µM calcium input, respectively. (B, C) Synaptic efficacy after 40 minutes of input ratio to pre-stimulus. Characteristics of dopamine- and calcium-dependent synaptic plasticity remain.
(0.43 MB EPS)

Table S1 Initial concentrations
(0.03 MB XLS)

Table S2 Enzymatic reactions
(0.04 MB XLS)

Table S3 Binding reactions
(0.02 MB XLS)

## References

[1] Hikosaka O (2005). Basal ganglia orient eyes to reward.. Journal of Neurophysiology.

[2] Doya K (2007). Reinforcement learning: Computational theory and biological mechanisms.. HFSP J.

[3] Balleine BW, Doya K, O'Doherty J, Sakagami M (2007). Current trends in decision making.. Ann N Y Acad Sci.

[4] Grace AA (2000). Gating of information flow within the limbic system and the pathophysiology of schizophrenia.. Brain Res Brain Res Rev.

[5] Nestler EJ (2001). Molecular basis of long-term plasticity underlying addiction.. Nat Rev Neurosci.

[6] Kelley AE (2004). Memory and addiction: shared neural circuitry and molecular mechanisms.. Neuron.

[7] Arbuthnott GW, Ingham CA, Wickens JR (2000). Dopamine and synaptic plasticity in the neostriatum.. J Anat.

[8] Calabresi P, Maj R, Pisani A, Mercuri NB, Bernardi G (1992). Long-term synaptic depression in the striatum: physiological and pharmacological characterization.. J Neurosci.

[9] Wickens JR, Begg AJ, Arbuthnott GW (1996). Dopamine reverses the depression of rat corticostriatal synapses which normally follows high-frequency stimulation of cortex in vitro.. Neuroscience.

[10] Reynolds JN, Wickens JR (2000). Substantia nigra dopamine regulates synaptic plasticity and membrane potential fluctuations in the rat neostriatum, in vivo.. Neuroscience.

[11] Calabresi P, Pisani A, Mercuri N, Bernardi G (1992). Long-term potentiation in the striatum is unmasked by removing the voltage-dependent magnesium block of NMDA receptor channels.. Eur J Neurosci.

[12] Svenningsson P, Nishi A, Fisone G, Girault JA, Nairn AC (2004). DARPP-32: an integrator of neurotransmission.. Annu Rev Pharmacol Toxicol.

[13] Greengard P, Nairn AC, Girault JA, Ouimet CC, Snyder GL (1998). The DARPP-32/protein phosphatase-1 cascade: a model for signal integration.. Brain Res Brain Res Rev.

[14] Halpain S, Girault JA, Greengard P (1990). Activation of NMDA receptors induces dephosphorylation of DARPP-32 in rat striatal slices.. Nature.

[15] Nishi A, Snyder GL, Greengard P (1997). Bidirectional regulation of DARPP-32 phosphorylation by dopamine.. J Neurosci.

[16] Nishi A, Bibb JA, Snyder GL, Higashi H, Nairn AC (2000). Amplification of dopaminergic signaling by a positive feedback loop.. Proc Natl Acad Sci USA.

[17] Nishi A, Bibb JA, Matsuyama S, Hamada M, Higashi H (2002). Regulation of DARPP-32 dephosphorylation at PKA- and Cdk5-sites by NMDA and AMPA receptors: distinct roles of calcineurin and protein phosphatase-2A.. J Neurochem.

[18] Nishi A, Watanabe Y, Higashi H, Tanaka M, Nairn AC (2005). Glutamate regulation of DARPP-32 phosphorylation in neostriatal neurons involves activation of multiple signaling cascades.. Proc Natl Acad Sci USA.

[19] Svenningsson P, Nairn AC, Greengard P (2005). DARPP-32 mediates the actions of multiple drugs of abuse.. The AAPS journal.

[20] Malinow R, Malenka RC (2002). AMPA receptor trafficking and synaptic plasticity.. Annu Rev Neurosci.

[21] Fernandez E, Schiappa R, Girault JA, Novère NL (2006). DARPP-32 is a robust integrator of dopamine and glutamate signals.. PLoS Comput Biol.

[22] Lindskog M, Kim M, Wikström MA, Blackwell KT, Kotaleski JH (2006). Transient calcium and dopamine increase PKA activity and DARPP-32 phosphorylation.. PLoS Comput Biol.

[23] Barbano PE, Spivak M, Flajolet M, Nairn AC, Greengard P (2007). A mathematical tool for exploring the dynamics of biological networks.. Proc Natl Acad Sci USA.

[24] Calabresi P, Centonze D, Gubellini P, Marfia GA, Bernardi G (1999). Glutamate-triggered events inducing corticostriatal long-term depression.. J Neurosci.

[25] Choi S, Lovinger DM (1997). Decreased probability of neurotransmitter release underlies striatal long-term depression and postnatal development of corticostriatal synapses.. Proc Natl Acad Sci USA.

[26] Reynolds JN, Hyland BI, Wickens JR (2001). A cellular mechanism of reward-related learning.. Nature.

[27] Reynolds JNJ, Wickens JR (2002). Dopamine-dependent plasticity of corticostriatal synapses.. Neural networks : the official journal of the International Neural Network Society.

[28] Calabresi P, Giacomini P, Centonze D, Bernardi G (2000). Levodopa-induced dyskinesia: a pathological form of striatal synaptic plasticity?. Ann Neurol.

[29] Centonze D, Picconi B, Gubellini P, Bernardi G, Calabresi P (2001). Dopaminergic control of synaptic plasticity in the dorsal striatum.. Eur J Neurosci.

[30] Carter AG, Sabatini BL (2004). State-dependent calcium signaling in dendritic spines of striatal medium spiny neurons.. Neuron.

[31] Akopian G, Musleh W, Smith R, Walsh JP (2000). Functional state of corticostriatal synapses determines their expression of short- and long-term plasticity.. Synapse.

[32] Charpier S, Deniau JM (1997). In vivo activity-dependent plasticity at cortico-striatal connections: evidence for physiological long-term potentiation.. Proc Natl Acad Sci USA.

[33] Charpier S, Mahon S, Deniau JM (1999). In vivo induction of striatal long-term potentiation by low-frequency stimulation of the cerebral cortex.. Neuroscience.

[34] Spencer JP, Murphy KP (2000). Bi-directional changes in synaptic plasticity induced at corticostriatal synapses in vitro.. Experimental brain research Experimentelle Hirnforschung Expérimentation cérébrale.

[35] Gould TD, Manji HK (2005). DARPP-32: A molecular switch at the nexus of reward pathway plasticity.. Proc Natl Acad Sci USA.

[36] Greengard P (2001). The neurobiology of dopamine signaling.. Biosci Rep.

[37] Greengard P, Allen PB, Nairn AC (1999). Beyond the dopamine receptor: the DARPP-32/protein phosphatase-1 cascade.. Neuron.

[38] Håkansson K, Lindskog M, Pozzi L, Usiello A, Fisone G (2004). DARPP-32 and modulation of cAMP signaling: involvement in motor control and levodopa-induced dyskinesia.. Parkinsonism Relat Disord.

[39] Rakhilin SV, Olson PA, Nishi A, Starkova NN, Fienberg AA (2004). A network of control mediated by regulator of calcium/calmodulin-dependent signaling.. Science.

[40] Hersch SM, Ciliax BJ, Gutekunst CA, Rees HD, Heilman CJ (1995). Electron microscopic analysis of D1 and D2 dopamine receptor proteins in the dorsal striatum and their synaptic relationships with motor corticostriatal afferents.. J Neurosci.

[41] Kawaguchi Y (1997). Neostriatal cell subtypes and their functional roles.. Neurosci Res.

[42] Sivakumaran S, Hariharaputran S, Mishra J, Bhalla US (2003). The Database of Quantitative Cellular Signaling: management and analysis of chemical kinetic models of signaling networks.. Bioinformatics.

[43] Markevich NI, Hoek JB, Kholodenko BN (2004). Signaling switches and bistability arising from multisite phosphorylation in protein kinase cascades.. J Cell Biol.

[44] Esteban JA, Shi SH, Wilson C, Nuriya M, Huganir RL (2003). PKA phosphorylation of AMPA receptor subunits controls synaptic trafficking underlying plasticity.. Nat Neurosci.

[45] Ehlers MD (2000). Reinsertion or degradation of AMPA receptors determined by activity-dependent endocytic sorting.. Neuron.

[46] Hempel CM, Vincent P, Adams SR, Tsien RY, Selverston AI (1996). Spatio-temporal dynamics of cyclic AMP signals in an intact neural circuitm.. Nature.

[47] Mons N, Cooper DM (1994). Selective expression of one Ca(2+)-inhibitable adenylyl cyclase in dopaminergically innervated rat brain regions.. Brain Res Mol Brain Res.

[48] Guillou JL, Nakata H, Cooper DM (1999). Inhibition by calcium of mammalian adenylyl cyclases.. J Biol Chem.

[49] Cooper DMF (2003). Molecular and cellular requirements for the regulation of adenylate cyclases by calcium.. Biochem Soc Trans.

[50] Hudmon A, Schulman H (2002). Structure-function of the multifunctional ca2+/calmodulin-dependent protein kinase ii.. Biochem J.

[51] Hudmon A, Schulman H (2002). Neuronal ca2+/calmodulin-dependent protein kinase ii: the role of structure and autoregulation in cellular function.. Annu Rev Biochem.

[52] Bradshaw JM, Kubota Y, Meyer T, Schulman H (2003). An ultrasensitive Ca2+/calmodulin-dependent protein kinase II-protein phosphatase 1 switch facilitates specificity in postsynaptic calcium signaling.. Proc Natl Acad Sci USA.

[53] Janssens V, Jordens J, Stevens I, Hoof CV, Martens E (2003). Identification and functional analysis of two Ca2+-binding EF-hand motifs in the B″/PR72 subunit of protein phosphatase 2A.. J Biol Chem.

[54] Usui H, Inoue R, Tanabe O, Nishito Y, Shimizu M (1998). Activation of protein phosphatase 2A by cAMP-dependent protein kinase-catalyzed phosphorylation of the 74-kDa B″ (delta) regulatory subunit in vitro and identification of the phosphorylation sites.. FEBS Lett.

[55] Desdouits F, Siciliano JC, Greengard P, Girault JA (1995). Dopamine- and cAMP-regulated phosphoprotein DARPP-32: phosphorylation of Ser-137 by casein kinase I inhibits dephosphorylation of Thr-34 by calcineurin.. Proc Natl Acad Sci USA.

[56] Hemmings HC, Greengard P, Tung HY, Cohen P (1984). DARPP-32, a dopamine-regulated neuronal phosphoprotein, is a potent inhibitor of protein phosphatase-1.. Nature.

[57] Hemmings HC, Nairn AC, Greengard P (1984). DARPP-32, a dopamine- and adenosine 3′:5′-monophosphate-regulated neuronal phosphoprotein. II. Comparison of the kinetics of phosphorylation of DARPP-32 and phosphatase inhibitor 1.. J Biol Chem.

[58] Hemmings HC, Williams KR, Konigsberg WH, Greengard P (1984). DARPP-32, a dopamine- and adenosine 3′:5′-monophosphate-regulated neuronal phosphoprotein. I. Amino acid sequence around the phosphorylated threonine.. J Biol Chem.

[59] Liu F, Virshup DM, Nairn AC, Greengard P (2002). Mechanism of regulation of casein kinase I activity by group I metabotropic glutamate receptors.. J Biol Chem.

[60] Desdouits F, Cohen D, Nairn AC, Greengard P, Girault JA (1995). Phosphorylation of DARPP-32, a dopamine- and cAMP-regulated phosphoprotein, by casein kinase I in vitro and in vivo.. J Biol Chem.

[61] Desdouits F, Siciliano JC, Nairn AC, Greengard P, Girault JA (1998). Dephosphorylation of Ser-137 in DARPP-32 by protein phosphatases 2A and 2C: different roles in vitro and in striatonigral neurons.. Biochem J.

[62] King MM, Huang CY, Chock PB, Nairn AC, Hemmings HC (1984). Mammalian brain phosphoproteins as substrates for calcineurin.. J Biol Chem.

[63] Klee CB, Draetta GF, Hubbard MJ (1988). Calcineurin.. Adv Enzymol Relat Areas Mol Biol.

[64] Liu F, Ma XH, Ule J, Bibb JA, Nishi A (2001). Regulation of cyclin-dependent kinase 5 and casein kinase 1 by metabotropic glutamate receptors.. Proc Natl Acad Sci USA.

[65] Bibb JA (2003). Role of Cdk5 in neuronal signaling, plasticity, and drug abuse.. Neurosignals.

[66] Bibb JA, Snyder GL, Nishi A, Yan Z, Meijer L (1999). Phosphorylation of DARPP-32 by Cdk5 modulates dopamine signalling in neurons.. Nature.

[67] Nishi A, Snyder GL, Nairn AC, Greengard P (1999). Role of calcineurin and protein phosphatase-2A in the regulation of DARPP-32 dephosphorylation in neostriatal neurons.. J Neurochem.

[68] Kötter R (1994). Postsynaptic integration of glutamatergic and dopaminergic signals in the striatum.. Prog Neurobiol.

[69] Snyder GL, Allen PB, Fienberg AA, Valle CG, Huganir RL (2000). Regulation of phosphorylation of the GluR1 AMPA receptor in the neostriatum by dopamine and psychostimulants in vivo.. J Neurosci.

[70] Girault JA, Hemmings HC, Williams KR, Nairn AC, Greengard P (1989). Phosphorylation of DARPP-32, a dopamine- and cAMP-regulated phosphoprotein, by casein kinase II.. J Biol Chem.

[71] Li T, Chalifour LE, Paudel HK (2007). Phosphorylation of protein phosphatase 1 by cyclin-dependent protein kinase 5 during nerve growth factor-induced PC12 cell differentiation.. J Biol Chem.

[72] Bhalla US, Iyengar R (1999). Emergent properties of networks of biological signaling pathways.. Science.

[73] Castellani GC, Bazzani A, Cooper LN (2009). Toward a microscopic model of bidirectional synaptic plasticity.. Proc Natl Acad Sci USA.

[74] Snyder GL, Galdi S, Fienberg AA, Allen PB, Nairn AC (2003). Regulation of AMPA receptor dephosphorylation by glutamate receptor agonists.. Neuropharmacology.

[75] Calabresi P, Gubellini P, Centonze D, Picconi B, Bernardi G (2000). Dopamine and cAMP-regulated phosphoprotein 32 kDa controls both striatal long-term depression and long-term potentiation, opposing forms of synaptic plasticity.. J Neurosci.

[76] Day M, Wokosin D, Plotkin JL, Tian X, Surmeier DJ (2008). Differential excitability and modulation of striatal medium spiny neuron dendrites.. Journal of Neuroscience.

[77] Bonsi P, Pisani A, Bernardi G, Calabresi P (2003). Stimulus frequency, calcium levels and striatal synaptic plasticity.. Neuroreport.

[78] Kötter R, Wickens J (1995). Interactions of glutamate and dopamine in a computational model of the striatum.. Journal of computational neuroscience.

[79] Gonon F, Burie JB, Jaber M, Benoit-Marand M, Dumartin B (2000). Geometry and kinetics of dopaminergic transmission in the rat striatum and in mice lacking the dopamine transporter.. Prog Brain Res.

[80] Hoops S, Sahle S, Gauges R, Lee C, Pahle J (2006). COPASI–a COmplex PAthway SImulator.. Bioinformatics.

[81] Lisman J, Schulman H, Cline H (2002). The molecular basis of CaMKII function in synaptic and behavioural memory.. Nat Rev Neurosci.

[82] Hayer A, Bhalla US (2005). Molecular switches at the synapse emerge from receptor and kinase traffic.. PLoS Comput Biol.

[83] Lee HK, Barbarosie M, Kameyama K, Bear MF, Huganir RL (2000). Regulation of distinct AMPA receptor phosphorylation sites during bidirectional synaptic plasticity.. Nature.

[84] Bernard V, Somogyi P, Bolam JP (1997). Cellular, subcellular, and subsynaptic distribution of AMPA-type glutamate receptor subunits in the neostriatum of the rat.. J Neurosci.

[85] Deng YP, Xie JP, Wang HB, Lei WL, Chen Q (2007). Differential localization of the GluR1 and GluR2 subunits of the AMPA-type glutamate receptor among striatal neuron types in rats.. J Chem Neuroanat.

[86] Passafaro M, Piëch V, Sheng M (2001). Subunit-specific temporal and spatial patterns of AMPA receptor exocytosis in hippocampal neurons.. Nat Neurosci.

[87] Tan CH, He X, Yang J, Ong WY (2006). Changes in AMPA subunit expression in the mouse brain after chronic treatment with the antidepressant maprotiline: a link between noradrenergic and glutamatergic function?. Experimental brain research Experimentelle Hirnforschung Expérimentation cérébrale.

[88] Swanson GT, Kamboj SK, Cull-Candy SG (1997). Single-channel properties of recombinant AMPA receptors depend on RNA editing, splice variation, and subunit composition.. J Neurosci.

[89] D'Alcantara P, Schiffmann SN, Swillens S (2003). Bidirectional synaptic plasticity as a consequence of interdependent Ca2+-controlled phosphorylation and dephosphorylation pathways.. Eur J Neurosci.

[90] Castellani GC, Quinlan EM, Bersani F, Cooper LN, Shouval HZ (2005). A model of bidirectional synaptic plasticity: from signaling network to channel conductance.. Learn Mem.

[91] Derkach V, Barria A, Soderling TR (1999). Ca2+/calmodulin-kinase II enhances channel conductance of alpha-amino-3-hydroxy-5-methyl-4-isoxazolepropionate type glutamate receptors.. Proc Natl Acad Sci USA.

[92] Roche KW, O'Brien RJ, Mammen AL, Bernhardt J, Huganir RL (1996). Characterization of multiple phosphorylation sites on the AMPA receptor GluR1 subunit.. Neuron.

[93] Banke TG, Bowie D, Lee H, Huganir RL, Schousboe A (2000). Control of GluR1 AMPA receptor function by cAMP-dependent protein kinase.. J Neurosci.

[94] Lengyel I, Voss K, Cammarota M, Bradshaw K, Brent V (2004). Autonomous activity of CaMKII is only transiently increased following the induction of long-term potentiation in the rat hippocampus.. Eur J Neurosci.

[95] Bito H, Takemoto-Kimura S (2003). Ca(2+)/CREB/CBP-dependent gene regulation: a shared mechanism critical in long-term synaptic plasticity and neuronal survival.. Cell Calcium.

[96] Hu SC, Chrivia J, Ghosh A (1999). Regulation of CBP-mediated transcription by neuronal calcium signaling.. Neuron.

[97] Calabresi P, Saulle E, Marfia GA, Centonze D, Mulloy R (2001). Activation of metabotropic glutamate receptor subtype 1/protein kinase C/mitogen-activated protein kinase pathway is required for postischemic long-term potentiation in the striatum.. Mol Pharmacol.

[98] Paul S, Snyder GL, Yokakura H, Picciotto MR, Nairn AC (2000). The Dopamine/D1 receptor mediates the phosphorylation and inactivation of the protein tyrosine phosphatase STEP via a PKA-dependent pathway.. J Neurosci.

[99] Nairn AC, Svenningsson P, Nishi A, Fisone G, Girault JA (2004). The role of DARPP-32 in the actions of drugs of abuse.. Neuropharmacology.

[100] Wilson CJ, Kawaguchi Y (1996). The origins of two-state spontaneous membrane potential fluctuations of neostriatal spiny neurons.. J Neurosci.

[101] Gerfen CR (2000). Molecular effects of dopamine on striatal-projection pathways.. Trends Neurosci.

[102] Gerfen CR, Engber TM, Mahan LC, Susel Z, Chase TN (1990). D1 and D2 dopamine receptor-regulated gene expression of striatonigral and striatopallidal neurons.. Science.

[103] Schultz W, Dayan P, Montague PR (1997). A neural substrate of prediction and reward.. Science.

[104] Nakano T, Yoshimoto J, Wickens J, Doya K (2009). Calcium responses model in striatum dependent on timed input sources.. International Conference of Artificial Neurai Networks.

[105] Hamada M, Hendrick JP, Ryan GR, Kuroiwa M, Higashi H (2005). Nicotine regulates DARPP-32 (dopamine- and cAMP-regulated phosphoprotein of 32 kDa) phosphorylation at multiple sites in neostriatal neurons.. J Pharmacol Exp Ther.

[106] Ahmed SH (2004). Neuroscience. Addiction as compulsive reward prediction.. Science.

[107] Bezprozvanny I, Watras J, Ehrlich BE (1991). Bell-shaped calcium-response curves of Ins(1,4,5)P3- and calcium-gated channels from endoplasmic reticulum of cerebellum.. Nature.

